# Early-onset gastrointestinal amyloid A amyloidosis without renal involvement in a patient with RA–pSpA overlap: A case report

**DOI:** 10.1097/MD.0000000000046291

**Published:** 2025-11-28

**Authors:** Hyeok Chan Kwon

**Affiliations:** a Department of Internal Medicine, Division of Rheumatology, Dankook University College of Medicine, Dongnam-gu, Cheonan-si, Chungcheongnam-do, Republic of Korea.

**Keywords:** amyloid A protein, amyloidosis, arthritis, case reports, gastrointestinal tract, HLA-B27 antigen, rheumatoid, spondyloarthritis

## Abstract

**Rationale:**

Amyloid A (AA) amyloidosis involving the gastrointestinal (GI) tract is a rare but potentially life-threatening complication of chronic inflammatory rheumatic diseases. Although typically associated with long-standing rheumatoid arthritis (RA), it is rarely reported in the context of RA–peripheral spondyloarthritis (pSpA) overlap syndrome. We report a rare case of early-onset GI-predominant AA amyloidosis without renal involvement in a patient with overlapping RA and HLA-B27–positive pSpA.

**Patient concerns:**

A 52-year-old male presented with progressive arthritis involving the knees, ankles, and toes. Despite RA treatment, inflammatory markers remained elevated. He later developed anterior uveitis and gastrointestinal symptoms including abdominal pain and diarrhea.

**Diagnoses:**

He fulfilled classification criteria for both RA and pSpA. Endoscopic biopsy revealed amyloid A deposits in the stomach and duodenum.

**Interventions:**

Intravenous tocilizumab was initiated every 4 weeks.

**Outcomes:**

After 2 months of tocilizumab treatment, GI symptoms markedly improved, inflammatory markers and serum albumin normalized, and imaging demonstrated resolution of bowel wall edema. Serum amyloid A levels also markedly decreased to within the normal range. The patient achieved sustained remission of articular and gastrointestinal manifestations at 6-month follow-up.

**Lessons:**

This case illustrates early GI-dominant AA amyloidosis without renal involvement in RA–pSpA overlap. The coexistence of intense inflammatory responses and mucosal immune activation may predispose the GI tract to early amyloid deposition, emphasizing the need for active surveillance and early endoscopic evaluation in patients presenting with unexplained GI symptoms.

## 1. Introduction

Amyloid A (AA) amyloidosis, characterized by the extracellular deposition of fibrils derived from the serum amyloid A protein (SAA), is a serious complication of chronic inflammatory diseases. Although less common than renal involvement, gastrointestinal (GI) involvement in amyloidosis can be clinically significant.^[1]^ In systemic AA amyloidosis, renal involvement is almost universal, reported in up to 97% of patients, whereas gastrointestinal manifestations are uncommon, accounting for only about 3% to 4% of biopsy-proven systemic cases. Notably, isolated GI involvement without renal disease has been documented only in exceedingly rare instances.^[1,2]^ Rheumatoid arthritis (RA) is the leading cause of AA amyloidosis, whereas spondyloarthritis (SpA), including ankylosing spondylitis (AS), is also implicated less frequently.^[3]^ Interleukin (IL)-6, a key driver of SAA synthesis, plays a central role in the pathogenesis of AA amyloidosis, and its inhibition has demonstrated therapeutic potential.^[4]^

Herein, we report a rare case of early onset GI-predominant AA amyloidosis without renal involvement in a patient with overlapping RA and human leukocyte antigen B27 (HLA-B27)-positive peripheral SpA (pSpA), suggesting that amplified IL-6–mediated inflammation and overlapping immune mechanisms may contribute to atypical organ involvement.

## 2. Case presentation

A 52-year-old male presented with a 5-month history of left knee arthralgia. The patient initially received antibiotic therapy at an external hospital for presumed infectious arthritis. However, his symptoms did not improve, prompting referral to our facility for further evaluation. During hospitalization, the pain extended to the right knee, accompanied by the development of arthralgia in both ankles and toes. At presentation, the patient was afebrile and had stable vital signs. The patient had a history of type 2 diabetes mellitus managed with oral hypoglycemic agents. He had a smoking history of 20 pack-years but had quit smoking at the time of presentation. The patient reported no relevant family medical history. At presentation, the patient’s vital signs were stable: blood pressure 118/78 mm Hg, heart rate 85 beats/min, respiratory rate 20 breaths/min, and body temperature 36.0 °C. Upon referral to the rheumatology department, a physical examination revealed warmth, swelling, and tenderness in both knees, with similar findings in both ankles and metatarsophalangeal joints.

Laboratory results were as follows: white blood cells, 15.39 × 10^3^/μL; neutrophils, 12.99 × 10^3^/μL; hemoglobin, 11.1 g/dL; platelets, 430 × 10^3^/μL; C-reactive protein (CRP), 8.61 mg/dL; erythrocyte sedimentation rate (ESR), 120 mm/h; serum creatinine, 0.65 mg/dL; serum albumin, 3.6 g/dL; uric acid, 3.0 mg/dL; rheumatoid factor (RF), 31.7 IU/mL (normal range: 0–15); and anti-cyclic-citrullinated antibodies (anti-CCP Ab), 7.9 U/mL (normal level < 5). Proteinuria was not observed on urinalysis. All bacterial cultures and tuberculosis tests yielded negative results. Power Doppler ultrasonography of the left knee demonstrated hypoechoic synovial thickening with increased vascular flow in the suprapatellar recess, indicating active synovitis. The patient met the 2010 American College of Rheumatology/European League Against Rheumatism classification criteria for RA, scoring 7 points based on definite synovitis, positive RF and anti-CCP Abs, elevated CRP and ESR levels, and symptom duration exceeding 6 weeks. Accordingly, a diagnosis of seropositive RA was established.^[5]^ Initial treatment included methotrexate, sulfasalazine, and non-steroidal anti-inflammatory drugs, with outpatient follow-up. Although symptoms partially improved, inflammatory markers remained elevated and did not normalize.

During the follow-up, the patient developed bilateral anterior uveitis and tested positive for HLA-B27, although sacroiliitis and inflammatory back pain were not detected on sacroiliac joint radiography. Based on the Assessment of Spondyloarthritis International Society classification criteria, the presence of HLA-B27 positivity, uveitis, and peripheral arthritis, predominantly affecting the lower extremities, is consistent with pSpA.^[6]^ The patient was considered to have HLA-B27-positive RA with pSpA features, suggesting an overlap between RA and pSpA.

Despite dose escalation of disease-modifying antirheumatic drugs (DMARDs), the CRP levels remained elevated 5 months after treatment initiation. The patient developed abdominal pain, diarrhea, and anorexia. Laboratory findings were as follows: CRP, 4.46 mg/dL; ESR, 56 mm/h; serum albumin, 2.4 g/dL; and total protein, 4.7 g/dL. Serum creatinine levels remained normal and no proteinuria was observed. Given the patient’s HLA-B27 positivity and the possibility of underlying inflammatory bowel disease, esophagogastroduodenoscopy and colonoscopy were performed. Esophagogastroduodenoscopy revealed generalized mucosal edema and erythema with a mosaic-like pattern in the gastric mucosa. Conservative treatment led to temporary improvement, and the patient was discharged after undergoing an endoscopic biopsy.

**Figure 1.**
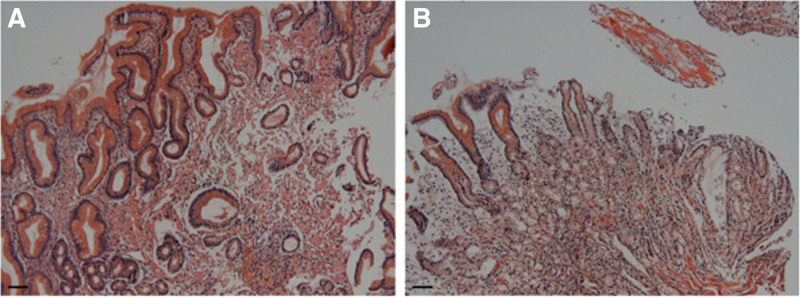
Histopathological findings of the gastric biopsy specimen stained with Congo red (×100). (A) Congophilic amorphous deposits are diffusely distributed throughout the lamina propria, predominantly between the gastric glands. (B) Amyloid deposition is also noted in the submucosal region, especially surrounding small blood vessels. A 20 µm black scale bar is shown.

**Figure 2.**
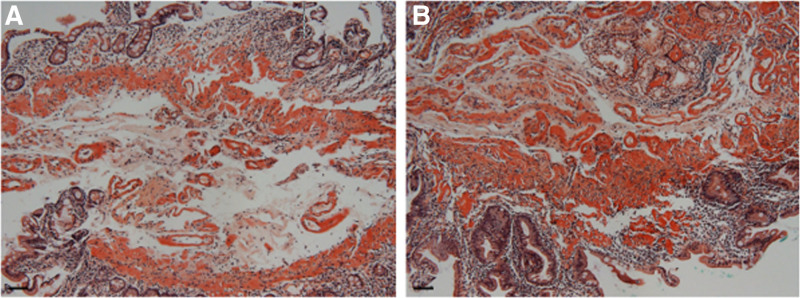
Histopathological findings of the duodenal biopsy specimen stained with Congo red (×100). (A) Dense congophilic deposits are visible along the mucosal surface and within the villous cores of the duodenal mucosa. (B) Prominent perivascular amyloid deposits are observed in the submucosa. A 20 µm black scale bar is shown.

**Figure 3.**
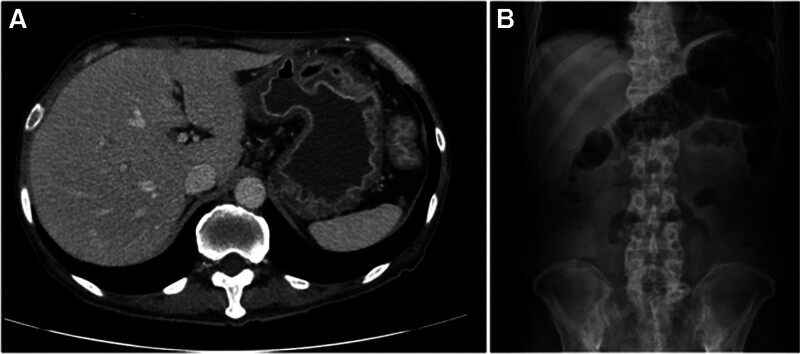
Abdominopelvic-computed tomography (AP-CT) and upright abdominal radiograph findings in a patient with gastrointestinal amyloidosis. (A) AP-CT demonstrates diffuse concentric thickening of the entire gastric wall with mucosal enhancement. (B) Upright radiograph shows multiple air-fluid levels and small bowel dilatation, suggestive of paralytic ileus.

**Figure 4.**
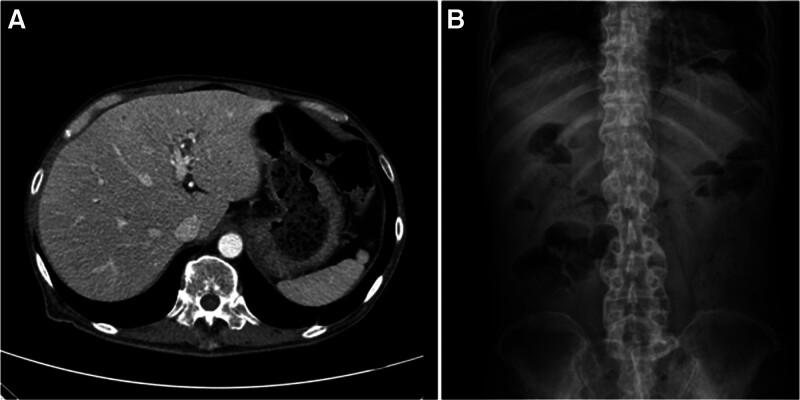
Follow-up imaging performed 2 months after tocilizumab initiation for gastrointestinal amyloidosis. (A) Compared to the previous study, gastric wall thickening has improved and the gastric lumen is more distended with visible intraluminal contents. (B) Resolution of previous air-fluid levels and decreased small bowel dilatation indicate radiologic improvement in gastrointestinal transit.

The abdominal symptoms recurred with poor oral intake and persistent abdominal pain, diarrhea, nausea, and vomiting for several days. Histopathological review of the prior endoscopic biopsy specimens confirmed gastroduodenal amyloidosis involving the stomach and duodenum (Figs. 1 and 2). The colonic mucosa exhibited chronic active inflammation with erosion, but no definitive histopathological evidence of amyloidosis was observed. Upon readmission, contrast-enhanced abdominopelvic computed tomography (AP-CT) and abdominal radiography revealed diffuse edematous wall thickening of the stomach, small intestine, and large intestine, along with paralytic ileus (Fig. 3). Serum albumin levels further declined to 1.8 g/dL, and serum amyloid A (SAA) levels were markedly elevated to 300 mg/L (reference range: 0–11.0 mg/L). The patient was diagnosed with amyloidosis 1 year and 6 months after the initial onset of joint symptoms. Given the GI manifestations, oral DMARDs were discontinued, which led to the exacerbation of joint symptoms. Considering the clinical situation, biologic therapy with intravenous tocilizumab was initiated at 4-week intervals. The patient was hospitalized for 2 months to receive intensive conservative treatment. Follow-up AP-CT and abdominal radiography at 2 months post-biologic therapy demonstrated resolution of the previous bowel wall edema and improvement of ileus (Fig. 4). The patient’s GI symptoms improved, with restoration of oral intake and a marked reduction in joint pain. Laboratory tests revealed normal serum albumin (3.5 g/dL), CRP (0.2 mg/dL), and SAA (7.9 mg/L) levels. At the latest follow-up, the patient continued regular outpatient therapy with tocilizumab, with sustained clinical remission of the articular and GI manifestations for 6 months. The chronological sequence of clinical manifestations, diagnostic findings, and therapeutic interventions is summarized in Table 1.

**Table 1:** Timeline of clinical events, diagnostic findings, and therapeutic interventions in a patient with RA–pSpA overlap presenting with gastrointestinal amyloidosis.

Time from initial symptom onset	Clinical events/findings	Key interventions	Outcome/comments
Month–5	Onset of left kneearthralgia	Empiric antibiotics at local hospital (suspected infection)	No improvement
Month 0 (hospital presentation)	Progression to polyarthritis (ankles, & toes); afebrile; CRP 8.61mg/dL, ESR 120 mm/h	Diagnosed with seropositive RA (RF positive, anti-CCP Ab positive)	Diagnosis of RA confirmed
Month 1	Persistent arthritis despite DMARDs	Methotrexate, sulfasalazine	Partial improvement
Month 5	Bilateral anterior uveitis; HLA-B27 positive; no sacroiliitis → RA–pSpAoverlap confirmed	Continued DMARDs	Inflammation persists
Month 5–6 (after pSpA diagnosis)	New GI symptoms: abdominal pain,diarrhea; Alb 2.4 g/dL, CRP 4.46 mg/dL	Endoscopy + biopsy → gastroduodenal AA amyloidosis confirmed	GI amyloidosis diagnosed; renal function normal
Month 6	Recurrent GI symptoms and paralytic ileus (Alb 1.8 g/dL, SAA 300 mg/L)	IV TCZ started monthly	Clinical & radiologic improvement
Month 8 (2 months after TCZ)	Alb 3.5 g/dL, SAA 7.9 mg/L; bowel wall edema resolved	Continued TCZ treatment	Stable, no recurrence
Month 12 (6 months follow-up)	Sustained remission of GI and joint symptoms	Maintenance TCZ treatment	Sustained remission; no recurrence at the latest follow-up.

AA = amyloid A, Alb = albumin, anti-CCP Ab = anti-cyclic citrullinated peptide antibody, CRP = C-reactive protein, DMARDs = disease-modifying antirheumatic drugs, ESR = erythrocyte sedimentation rate, GI = gastrointestinal, HLA-B27 = human leukocyte antigen-B27, IV = intravenous, pSpA = peripheral spondyloarthritis, RA = rheumatoid arthritis, RF = rheumatoid factor, SAA = serum amyloid A, TCZ = tocilizumab.

## 3. Discussion

AA amyloidosis is a systemic form of amyloidosis caused by the extracellular deposition of amyloid fibrils derived from the acute-phase reactant SAA protein, which is excessively produced in the liver under chronic inflammatory conditions. The kidneys are the most commonly affected organs, with over 97% of patients presenting with proteinuria and impaired renal function.^[1]^

Given the chronic inflammatory nature of rheumatic diseases, AA amyloidosis can lead to serious complications. Persistent elevation of SAA levels drives amyloid fibril deposition within tissues, and genetic susceptibility has been associated with AA amyloidosis development. Patients with RA are specifically at risk of disease progression.^[7]^ Although rare, AA amyloidosis has been reported in AS, where it may significantly affect clinical outcomes.^[4]^

Coexistence of RA and SpA is uncommon. However, one study reported that up to 6.6% of patients with RA may be positive for HLA-B27, suggesting that these diseases are not mutually exclusive but may coexist and may share overlapping clinical features.^[8]^ In the present case, the patient was initially diagnosed with RA based on the presence of specific autoantibodies. During follow-up, the patient developed uveitis and tested positive for HLA-B27, leading to the diagnosis of pSpA. As sacroiliitis was not detected on imaging, the final diagnosis was RA–pSpA overlap syndrome. Such overlap may represent a distinct inflammatory state, differing from classical RA or SpA.

Notably, our patient developed AA amyloidosis within 2 years of the onset of joint symptoms, whereas previous studies have reported a mean latency of 17 to 19 years for secondary AA amyloidosis.^[1,9]^ As GI symptoms were predominant, endoscopic evaluations were initially conducted to assess HLA-B27-associated inflammatory bowel disease. However, histological examination unexpectedly confirmed GI AA amyloidosis.

The RA–pSpA overlap may result in the convergence of the Th1/Th17-mediated immune response typical of RA and the IL-23/IL-17 axis and innate immunity characteristic of SpA. This combined inflammatory milieu may increase the production of proinflammatory cytokines such as IL-6, tumor necrosis factor-alpha, and IL-17, potentially accelerating amyloid deposition.^[8,10]^

Importantly, repeated assessments revealed no evidence of renal impairment or proteinuria. This contrasts with the typical presentation of systemic AA amyloidosis, in which renal involvement is observed in > 97% of cases, whereas isolated GI involvement is rare.^[1,11]^ One study reported isolated gastrointestinal amyloidosis in approximately 0.7 % of cases.^[2]^

Although the pathophysiology remains unclear, HLA-B27-positive individuals exhibit gut–vascular barrier and epithelial barrier dysfunction, leading to increased intestinal permeability, endotoxin translocation, and localized immune activation, particularly through the IL-23/IL-17 signaling pathway.^[12]^ Additionally, the gut-associated lymphoid tissue, which is rich in immune cells, responds more readily to chronic inflammation, resulting in early amyloid deposition in the GI tract.^[13]^ Therefore, in the context of the RA–pSpA overlap, this compounded inflammatory burden may have predisposed the gut to amyloid involvement over the kidneys.

From a therapeutic perspective, interleukin-6 (IL-6) plays a central role in driving hepatic synthesis of SAA, the precursor of AA amyloid fibrils. IL-6 receptor blockade with tocilizumab effectively suppresses SAA overproduction, thereby attenuating amyloidogenic pressure and potentially inducing stabilization or regression of existing amyloid deposits. Recent studies have demonstrated histologic regression of AA amyloid after long-term tocilizumab therapy and improvement in renal and systemic manifestations, supporting its potential as a disease-modifying therapy for AA amyloidosis.^[14,15]^

This report presents a rare case of early onset GI-dominant AA amyloidosis without renal involvement in a patient with concurrent RA and HLA-B27-positive pSpA. The combination of persistent systemic inflammation and HLA-B27-related mucosal immune activation suggests that the GI tract is the primary target organ for amyloid deposition. This finding highlights the importance of considering AA amyloidosis in the differential diagnosis of patients with atypical GI symptoms and overlapping inflammatory conditions.

## 4. Conclusion

This study reports a rare case of early onset GI AA amyloidosis without renal involvement in a patient with overlapping RA and HLA-B27-positive pSpA. The coexistence of intense inflammatory responses and mucosal immune activation may predispose the GI tract to early amyloid deposition, emphasizing the need for active surveillance and early endoscopic evaluation in patients presenting with unexplained GI symptoms.

## Acknowledgments

The author would like to thank the patient described in this case report and all individuals involved in collecting and organizing the medical records and data.

## Author contributions

**Conceptualization:** Hyeok Chan Kwon.

**Data curation:** Hyeok Chan Kwon.

**Formal analysis:** Hyeok Chan Kwon.

**Funding acquisition:** Hyeok Chan Kwon.

**Investigation:** Hyeok Chan Kwon.

**Methodology:** Hyeok Chan Kwon.

**Project administration:** Hyeok Chan Kwon.

**Resources:** Hyeok Chan Kwon.

**Supervision:** Hyeok Chan Kwon.

**Validation:** Hyeok Chan Kwon.

**Writing – original draft:** Hyeok Chan Kwon.

**Writing – review & editing:** Hyeok Chan Kwon.
